# Three injections of freeze‐dried platelet factor concentrate provide greater clinical response than single injection in the treatment of knee osteoarthritis

**DOI:** 10.1002/jeo2.70906

**Published:** 2026-09-29

**Authors:** Tadahiko Ohtsuru, Hiroto Hanai, Taro Akimori, Wataru Ando, Norimasa Nakamura, Stephen Lyman

**Affiliations:** ^1^ Omiya Knee Osteoarthritis Clinic Saitama Japan; ^2^ Ohtsuru Orthopedic Clinic Shizuoka Japan; ^3^ Department of Orthopedic Surgery Tokyo Women's Medical University School of Medicine Tokyo Japan; ^4^ Department of Orthopedic Surgery, Graduate School of Medicine Osaka University Osaka Japan; ^5^ Institute for Medical Science in Sports Osaka Health Sciences University Osaka Japan; ^6^ Department of Medical Education Kyushu University School of Medicine Fukuoka City Japan; ^7^ Department of Population Health Sciences Weill Cornell Medical College New York New York USA

**Keywords:** biologic healing enhancement, bone marrow aspirate concentrate, growth factors, platelet‐rich plasma, stem cell therapy

## Abstract

**Purpose:**

Single‐injection freeze‐dried platelet factor concentrate (PFC‐FD) has been shown to be safe and effective in the treatment of early knee osteoarthritis (OA) with clear advantages over other PRP processing methods. No previous clinical investigations have examined multiple PFC‐FD doses. The aim of this study was to assess the safety and effectiveness of one versus three PFC‐FD injections in patients with knee OA.

**Methods:**

Patients with Kellgren–Lawrence (KL) Grade I–IV knee OA were prospectively recruited via shared decision‐making based on patient preference to receive either one (*n* = 1054) or three (*n* = 317) PFC‐FD injections. Patients completed a pre‐injection knee injury and OA outcomes survey (KOOS). Post‐injection, patients completed the KOOS at 1, 3, 6 and 12 months. Adverse events were documented. Response to treatment was assessed using the Outcome Measures in Rheumatology (OMERACT)‐Osteoarthritis Research Society International responder criteria. Multivariable modelling assessed treatment effectiveness, adjusting for patient differences.

**Results:**

The three‐injection cohort demonstrated a small advantage in all KOOS subscales except quality of life (QOL) at 1 month (2.4–4.0 points, *p* < 0.0001–0.004), but this advantage disappeared by 3 months. When only early OA (KLI–II) was considered, the three‐injection cohort demonstrated higher improvement at 1, 3 and/or 6 months for most KOOS subscales. No such differences were observed for advanced OA patients. Multivariable analysis demonstrates that three‐injection patients were more likely to achieve responder status for KOOS‐Pain and KOOS‐activities of daily living (ADL) at 12 months (70.9% vs. 60.9%, *p* = 0.02 and 55.7% vs. 47.3%, *p* = 0.009, respectively). Overall patient‐level adverse events were higher in the three‐injection group (21.3% vs. 6.9%; *p* < 0.001), though adverse events per injection did not differ between groups (one‐injection 6.9%, three‐injection 8.6%; *p* = 0.19).

**Conclusion:**

Based on non‐randomized allocation, a three‐injection PFC‐FD treatment protocol demonstrates a higher likelihood of clinical response compared to a one‐injection protocol, particularly for relief of early knee OA symptoms, without evidence of an adverse immune response.

**Level of Evidence:**

Level II, prognostic prospective cohort study (patients enroled at different stages of disease progression).

**Abbreviations**
ADL: activities of daily living
ADSCs: adipose‐derived mesenchymal stem cells
AMSCs: amniotic‐derived mesenchymal stem cells
BMI: body mass index
BMSCs: bone marrow‐derived mesenchymal stem cells
HA: hyaluronic acid
IQR: interquartile range
K–L: Kellgren–Lawrence
KOOS: Knee injury and Osteoarthritis Outcomes Survey
NSAIDs: non‐steroidal anti‐inflammatory medications
OA: osteoarthritis
OARSI: Osteoarthritis Research Society International
PBS: phosphate‐buffered saline
PFC: platelet factor concentrate
PFC‐FD: freeze‐dried platelet factor concentrate
PROMs: patient‐reported outcome measures
PRP: platelet‐rich plasma
QOL: quality of life
RCTs: randomized controlled trials
TKA: total knee arthroplasty

## INTRODUCTION

Knee osteoarthritis (OA) has emerged as one of the chief limitations on mobility and quality of life (QOL) in older adults [5, 8, 12]. While the definitive treatment for advanced knee OA remains the modern condylar total knee arthroplasty (TKA) since its introduction in the 1970s [16], there is a continued and pressing need for non‐operative therapies in the management of early‐ to late‐stage knee OA. For non‐operative knee OA management, the Osteoarthritis Research Society International (OARSI) recommends these treatments: weight loss, physical therapy, non‐steroidal anti‐inflammatory medications (NSAIDs), steroid or hyaluronic acid (HA) injection, patient education and bracing [13, 27].

However, these management options are not expected to prevent the progression of knee OA, manage pain long‐term or improve knee function [5, 12]. Therefore, treatment options are needed for patients who have failed these non‐operative therapies but hope to avoid or cannot safely undergo TKA. In the United States, this group is estimated to be over 3.5 million patients who are living with long‐term pain and limited function, which may lead to workplace and social stresses [12]. Therefore, there remains a need for a non‐invasive option that is safe, provides adequate pain relief and slows the progression of knee OA [4].

While disease‐modifying pharmaceutical therapy has been successfully created, there have been a wide variety of biologic injection therapies developed. The primary injection modalities thus far developed and tested in humans include: platelet‐rich plasma (PRP), bone marrow‐derived mesenchymal stem cells (BMSCs), adipose‐derived mesenchymal stem cells (ADSCs) and amniotic‐derived mesenchymal stem cells (AMSCs) [3, 6, 7, 10, 18, 24, 25]. Each of these modalities has varying processing methods due to competing technologies used for cell processing and delivery. As such, the evidence for clinical effectiveness remains limited [6], though recent meta‐analyses seem to indicate clinical effectiveness of these therapies over placebo across injection modalities [15, 24].

In the specific context of orthobiologics, the European Society of Sports Traumatology, Knee Surgery and Arthroscopy (ESSKA) ORthoBIologics InitiaTive (ORBIT) consensus established that blood‐derived products, such as PRP, are a valid, first‐line injectable treatment option for the non‐operative management of early to moderate knee OA (Kellgren–Lawrence [K–L] Grades I–III) [11].

One modality that has demonstrated some clinical effectiveness for knee OA without compromising safety is freeze‐dried platelet factor concentrate (PFC‐FD) [16, 21]. This processing method has the advantages of being shelf‐stable and of releasing growth factors and cytokines directly into the joint rather than through intermediate platelet activity. The PFC‐FD preparation has previously been shown to provide clinically meaningful symptom relief in over 60% of patients when given in a single‐dose injection [16]. No prior studies have considered the potential for added benefit from multiple injections of PFC‐FC in the management of knee OA, though prior randomized controlled trials (RCTs) of PRP injections in one versus three‐dose protocols have demonstrated that multiple doses are more effective than single doses with minimal side effects [20]. The aim of this study was to compare the clinical and safety outcomes of one‐ versus three‐injection PFC‐FD in the setting of knee OA.

## MATERIALS AND METHODS

### Patients

This study was approved by the human research ethics committee of the Medical Corporation Katsujukai (approval number: 0006; Tokyo, Japan). Patients were prospectively recruited between August 2016 and January 2022 from 10 knee clinics across Japan. All patients were screened for eligibility. Only patients seeking their first knee injection were eligible for inclusion. Further eligibility criteria included age 18–95, X‐ray‐confirmed knee OA (KL Grade I or higher) and at least 6 months of knee OA symptoms. Exclusion criteria included prior knee surgery, any knee injection within the previous 30 days, history of anticoagulants or antiplatelet medications, inflammatory arthritis, auto‐allergic diseases, immunodeficiency or seropositivity for a chronic infectious disease (e.g., human immunodeficiency virus [HIV], hepatitis B/C, syphilis).

Patients who met the inclusion criteria were informed of the treatment options and invited to participate. They were informed that the evidence for safety and effectiveness is limited (one injection) or unknown (three injections). Those who provided their signed informed consent were enroled in this prospective cohort study. At this point, consulting surgeons guided the patients through an informed shared decision‐making process to choose their preferred treatment. This study complied with the Declaration of Helsinki and was approved by the local ethics board. STrengthening Reporting in OBservational studies in Epidemiology guidelines were adhered to for reporting.

### Interventions

All PFC‐FD processing was performed at a single cell culture processing laboratory (CellSource). The composition procedure for PFC‐FD is as follows. Whole blood (40 mL) was centrifuged (280×*g*) and rested for 10 min at room temperature. Top layer plasma was collected, centrifuged (1400×*g*) and again rested for 10 min at room temperature. The precipitated PRP was then collected. Moreover, 5.0 mL of phosphate‐buffered saline (PBS) and 1.5 mL of 2% (w/v) CaCl_2_ were added and suspended for 20 min at room temperature in order to compose the platelet‐derived factor concentrate (PFC). A 0.45 µm filter was used to remove cell components from the PFC, which was then freeze‐dried overnight. This cell processing protocol is based on the foundational work described by Araki et al in 2012 [1], which extracts platelet‐derived factors while replacing plasma with PBS and activating the platelets using CaCl_2_ to effectively remove fibrinogen to prevent gel formation and maximize growth factor release. Concentrations of the component growth factors and cytokines in this preparation were previously reported [16].

The solution was mixed in 6 mL of sterilized saline prior to administration of the intra‐articular injection. For single‐injection patients, this was administered one time. For 3‐injection patients, PFC‐FD for each injection dose was processed through independent blood draws; whole blood and injections were administered approximately 1 month apart. After treatment, all patients were instructed to adhere to a standardized regimen of self‐directed home physical therapy. Patients were permitted to seek independent physical therapist‐guided rehabilitation if they were not comfortable with the self‐directed regimen. No information was available regarding compliance with the self‐directed home therapy or therapist‐guided rehabilitation.

### Outcome measures

Patients completed the Japanese language knee injury and osteoarthritis outcomes score (J‐KOOS) pre‐injection and at 1, 3, 6, 12 and 24 month follow‐up timepoints [14] (Figure 1). All five subscales of the KOOS were considered: pain, symptoms, activities of daily living (ADL), QOL and sports and recreation. The Pain Visual Analogue Scale (VAS) was administered 1 week after each injection to evaluate residual pain as a potential adverse event after injection. Treatment effectiveness was measured using the Outcome Measures in Rheumatology (OMERACT)‐OARSI responder criteria [17]. Subjects were categorized by the severity of their OA using KL grade, and this was further combined to compare early (KL I–II) and more advanced OA (KL III–IV) for comparison [9].

**Figure 1:**
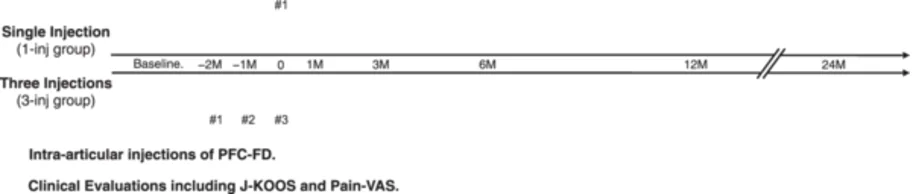
Treatment and follow‐up timeline for the one‐injection and three‐injection groups. J‐KOOS, Japanese language Knee injury and Osteoarthritis Outcomes Survey; PFC‐FD, freeze‐dried platelet factor concentrate; VAS, Visual Analogue Scale.

### Statistical methods

With 1054 one‐injection patients and 317 three‐injection patients enroled in this study, a compromise power analysis determined that this sample size provided 86% power to detect a 10% difference (e.g., 50% vs. 60%) in responder criteria status between groups (IBM SPSS Statistics 27 for Mac).

Standard descriptive statistics were calculated for all variables. The continuous variability normality assumption was tested using the Shapiro–Wilk test. Normally distributed continuous variables are reported with means and standard deviations, while non‐normal continuous variables are reported as median and interquartile range (IQR). Missing values were treated as missing. There was no missing data for age, sex or KL grade, while body mass index (BMI) was missing in <1% of patients.

Inferential statistics consisted of *χ* ^2^ tests for binary outcomes, while *t* test or an analogous non‐parametric equivalent was used for continuous outcomes. For multivariable analyses of the patient‐reported outcome measures (PROMs), a generalized linear model was built adjusting for age, sex, BMI and KL grade. A binary logistic regression model was also built, adjusting for the same covariates to assess variation in responder criteria achievement and adverse event risk by treatment and OA grade. Analyses were conducted using the SAS System, JMP and IBM SPSS Statistics 27.

No patients withdrew or were lost to follow‐up at 12 months, at which time 100% PROMs follow‐up was achieved. One‐month (93%), 3‐month (86%) and 6‐month (65%) PROMs follow‐ups were lower, though still acceptable by current best practices. However, just 40% of patients returned 2‐year PROMs.

No patients withdrew from the study. Patients who were lost to follow‐up were included in the analysis up until the point at which they were lost to follow‐up, after which their results were considered missing. Patients who failed the treatment protocol (sought intra‐articular injections not specified in the protocol or who sought surgical intervention within 12 months of PFC‐FD injection) were censored at the time of their additional treatment. These failures were compared between treatment protocol groups.

## RESULTS

A total of 1371 patients were enroled in this study and received baseline injections. A single injection was administered to 1054 patients and three injections to 317 patients. There were no significant differences between groups with regard to demographics or clinical characteristics, including baseline PROMs (Table 1).

**Table 1:** Baseline demographics between one‐ and three‐injection cohorts.

	One injection		Three injections		*p* value
	Mean	SD	Mean	SD	
Age in years	63.0	12.1	63.1	10.9	0.855
BMI	24.4	4.1	24.0	4.2	0.212
Baseline PROMs					
KOOS‐5	62.2	17.0	61.9	16.5	0.814
KOOS Pain	61.3	18.9	61.8	17.5	0.654
KOOS Symptoms	64.4	19.9	63.8	20.1	0.632
KOOS Activities of Daily Living	73.6	17.2	73.5	16.3	0.907
KOOS Sports and Recreation	42.4	26.2	41.4	26.7	0.524
KOOS Quality of Life	36.7	20.0	35.8	20.1	0.473
Pain VAS (100 mm)	52.5	25.4	52.8	25.3	0.808
Time to get up and go (s)	11.0	3.5	10.9	3.8	0.775

	***N***	**%**	***N***	**%**	***p* value**
Female sex	701	66.3	208	65.2	0.712
Kelgren–Lawrence grade					
I	97	9.2	24	7.6	0.109
II	299	28.4	86	27.1	
III	406	38.5	145	45.7	
IV	252	23.9	62	19.6	

Abbreviations: BMI, body mass index; KOOS, Knee injury and Osteoarthritis Outcomes Survey; PROMs, patient‐reported outcome measures; SD, standard deviation; VAS, Visual Analogue Scale.

A total of 77 patients sought off‐protocol injections within 12 months (65 [6.2%] of one‐injection patients and 12 [3.8%] of three‐injection patients, *p* = 0.107). Further, seven patients (four for TKA) opted for surgery within 12 months (5 one‐injection patients [0.5%] and 2 three‐injection patients [0.6%], *p* = 0.732).

## KOOS

All KOOS domain scores improved significantly at all follow‐up time‐points compared to baseline scores (Table 2). KOOS pain, ADL and symptom domains improved to 3 months, after which they plateaued. However, the quality of life (QOL) and sports and recreation domains continued to improve up to 24 months. Other subscales continued to improve out to 6 months, after which they plateaued at 12 months.

**Table 2:** KOOS domain comparisons.

	One injection	Three injections	Group comparison *p* value
	**Mean ± SD**	**Mean ± SD**	
KOOS‐5			
Baseline	62.0 ± 17.0	60.7 ± 17.0	0.22
1 month	71.0 ± 16.2	72.4 ± 16.2	0.19
3 months	73.9 ± 16.2	74.0 ± 16.9	0.93
6 months	75.1 ± 16.6	74.9 ± 17.2	0.84
12 months	75.8 ± 18.7	75.5 ± 18.4	0.80
24 months	77.8 ± 18.7	77.8 ± 19.2	0.99
ANOVA (timepoint) *p* value	<0.001	<0.001	
Activities of Daily Living			
Baseline	73.4 ± 17.2	72.5 ± 17.0	0.88
1 month	81.0 ± 15.2	82.5 ± 14.8	0.125
3 months	83.1 ± 15.0	83.6 ± 15.2	0.68
6 months	83.7 ± 15.3	83.6 ± 15.5	0.96
12 months	83.6 ± 17.1	83.7 ± 16.8	0.93
24 months	84.9 ± 17.0	85.7 ± 17.4	0.736
ANOVA (timepoint) *p* value	<0.001	<0.001	
Pain			
Baseline	61.0 ± 18.9	60.7 ± 17.8	0.813
1 month	71.7 ± 17.4	73.7 ± 16.7	0.081
3 months	74.8 ± 17.3	75.3 ± 17.9	0.64
6 months	76.2 ± 17.9	76.7 ± 18.0	0.733
12 months	76.8 ± 19.7	77.9 ± 18.5	0.377
24 months	79.1 ± 19.8	80.1 ± 19.1	0.707
ANOVA (timepoint) *p* value	<0.001	<0.001	
Quality of Life			
Baseline	36.3 ± 20.0	34.5 ± 20.1	0.157
1 month	47.8 ± 22.2	47.6 ± 21.8	0.862
3 months	52.5 ± 22.8	51.1 ± 22.4	0.401
6 months	55.2 ± 23.8	53.1 ± 23.7	0.262
12 months	57.1 ± 25.6	55.3 ± 24.3	0.275
24 months	61.7 ± 26.2	59.5 ± 27.4	0.515
ANOVA (timepoint) *p* value	<0.001	<0.001	
Symptoms			
Baseline	64.5 ± 20.0	62.0 ± 20.8	0.067
1 month	72.3 ± 17.8	73.3 ± 18.3	0.388
3 months	75.2 ± 17.5	74.9 ± 17.7	0.815
6 months	76.8 ± 17.7	76.2 ± 18.8	0.647
12 months	77.7 ± 19.3	76.2 ± 20.2	0.256
24 months	79.2 ± 19.1	79.7 ± 19.2	0.84
ANOVA (timepoint) *p* value	<0.001	<0.001	
Sports and Recreation			
Baseline	42.2 ± 26.1	39.5 ± 26.9	0.11
1 month	52.5 ± 27.1	54.3 ± 28.0	0.339
3 months	56.2 ± 27.5	56.1 ± 29.0	0.949
6 months	57.6 ± 27.7	57.3 ± 29.0	0.857
12 months	59.8 ± 30.3	58.3 ± 30.1	0.447
24 months	62.2 ± 30.6	59.4 ± 32.4	0.480
ANOVA (timepoint) *p* value	<0.001	<0.001	

Abbreviations: ANOVA, analysis of variance; KOOS, Knee injury and Osteoarthritis Outcomes Survey; SD, standard deviation.

After risk adjustment, patients receiving three injections demonstrated significant improvement over those receiving one injection at 1 month post‐treatment for all domains except QOL. These differences ranged from 2.4 to 4.0 points, which do not meet the minimal detectable change previously reported for these KOOS domains.

The change in scores was also significantly greater in the three‐injection group than in the one‐injection group at 1 month in all domains other than QOL (2.0–5.3 point difference). This advantage for the three‐injection group persisted out to 3 months for both the symptom and sports and recreation domains (2.4 and 3.3 point differences, respectively). Interestingly, the QOL domain was the only domain in which 3‐injection patients demonstrated significantly higher post‐injection scores than one‐injection patients among those with advanced OA (KL 3–4).

A sub‐analysis of KOOS domain change scores for one‐ versus three‐injections by KL grade (early KL1–2 vs. progressive KL3–4) adjusted for age, sex and BMI demonstrated superiority for three‐injections across all KOOS domains, most notably QOL, where a significant benefit was demonstrated out to 6 months. While not statistically significant (*p* = 0.051), this persisted out to 12 months.

In a repeated measures multivariable analysis adjusting for age, sex, BMI and KL OA grade, three‐injection patients had significantly more improvement in KOOS‐Pain (*p* = 0.024) and KOOS‐ADL (*p* = 0.010) scores than one‐injection patients.

### Responder rate

The percentage of patients meeting the OMERACT‐OARSI responder criteria was calculated at each time point (Table 3). Three‐injection patients were significantly more likely to achieve responder criteria at 1 year than one‐injection patients for both KOOS‐Pain (*p* < 0.001) and KOOS‐ADL (*p* = 0.013).

**Table 3:** Achievement of Outcome Measures in Rheumatology (OMERACT)‐OARSI criteria at 12 months.

KOOS domain	One injection	Three injections	*p* value
Pain	641 (60.9%)	224 (70.9%)	0.01
Symptoms	606 (57.6%)	194 (61.4%)	0.231
ADL	498 (47.3%)	176 (55.7%)	0.009
Sports and Recreation	574 (54.6%)	188 (59.5%)	0.122
QOL	689 (65.5%)	218 (6.9%)	0.249

Abbreviations: ADL, Activities of Daily Living; KOOS, Knee injury and Osteoarthritis Outcomes Survey; QOL, quality of life.

### Adverse events

As for adverse events, 73 (6.9%) of patients who received one injection suffered an adverse event, with 68 of these being pain and/or swelling that resolved within 2 weeks of injection with either no medical intervention or NSAIDs. On the other hand, 68 (21.3%) of three‐injection patients experienced 82 adverse events over the three injections, 76 of which were pain and/or swelling that resolved within 2 weeks of injection with NSAIDs or no treatment. As would be expected, there was a statistically significant increase in overall risk of adverse events in the three‐injection group (*p* < 0.001). However, when considered per injection, the adverse event frequency did not differ (6.9% vs. 8.6%; *p* = 0.19).

Serious adverse events included two cases of Herpes Zoster manifesting within 2 weeks of injection (one in each injection cohort) and one lacunar infarction. The lacunar infarction occurred in a 68‐year‐old female in the three‐injection cohort. She was hospitalized for 1 week, but still completed her third injection and follow‐up PROMs with a 1‐month delay.

## DISCUSSION

The main finding of this study is that a three‐injection PFC‐FD protocol provides a modest, though clear, advantage over a one‐injection PFC‐FD protocol. This is the first clinical study comparing the safety and effectiveness of one‐ versus three‐injection PFC‐FD in patients with knee OA. While both groups demonstrated significant improvements in all domains of the KOOS following treatment, the three‐injection group demonstrated a consistent but modest advantage in both KOOS‐pain and KOOS‐ADL improvement.

Both recent systematic reviews and recent meta‐analyses have demonstrated a consistent clinical benefits for biologic therapies in knee OA [1, 14, 23]. These findings add to this body of literature and further demonstrate that a dose‐response relationship may be at work, with three‐injection patients more likely to achieve the OMERACT/OARSI responder criteria for KOOS pain and ADL than one‐injection patients after adjustment for other confounding factors.

Again, as with prior studies [9, 15, 19], early knee OA patients appear to experience higher responsiveness to treatment than advanced knee OA patients. This is unsurprising given the advanced state of KL Grade IV patients' cartilage degeneration and the likelihood that a biologic process from PFC‐FD injection could have a meaningful clinical effect on a patient's symptoms.

With regard to pain relief, three‐injection patients were more likely to achieve KOOS‐pain MCIC at 12 months. This may reflect some preliminary evidence that sustained pain relief in the setting of knee OA may disrupt catastrophizing, or the propensity for patients in chronic pain to exaggerate or overly focus on the fear of continued pain. Unfortunately, patients were not queried about pain catastrophizing, so this assessment is purely speculative as to what this finding means, but it may be an area for future research.

Ultimately, these biologic injections, including PFC‐FD, should be seen as bridge therapies, which help delay the need for definitive surgical intervention with knee arthroplasty. Given that demand for knee arthroplasty has been increasing [21] and the age at which arthroplasty is being received has been declining [25], therapies that can delay the need for knee arthroplasty should be considered when possible. Reducing demand in the near term will not only save health system resources, but will also likely reduce the need for revision knee arthroplasty, which is also growing at alarming rates due to the large increases in primary implantations [6]. Reducing the likelihood that a patient will need a revision knee arthroplasty has substantial quality of life consequences [2].

Both one‐ and three‐injection PFC‐FD appear to be safe and effective therapy options for knee OA, particularly in those with earlier disease. The apparent clinical outcome advantages of three injections versus one injection need to be weighed against the burden for the patient and health system.

A final consideration is the clear advantages of PFC‐FD over on‐site PRP processing. The PFC‐FD processing allows for a shelf‐stable biologic injection option, which creates clear efficiency gains. Further, the PFC‐FD process extracts the available growth factors and cytokines in solution rather than the platelets in PRP injections releasing these growth factors and cytokines after injection and in unknown doses.

As with all biologic therapy research conducted in Japan, randomization was not possible due to the current regulatory environment. As such, blinding and completely balanced treatment groups were not achievable. Fortunately, risk adjustment in the multivariable modelling was able to adjust for differences in the patient populations. Due to the large number of patients, cartilage imaging in this analysis was infeasible, but these safety and outcomes findings add to the body of knowledge regarding the use of biologic therapies for knee OA.

In conclusion, a non‐randomized three‐injection PFC‐FD treatment protocol demonstrates a higher likelihood of clinical response compared to a one‐injection PFC‐FD treatment protocol for relief of knee OA symptoms without evidence of an adverse immune response. The clinical effectiveness is most apparent and sustained in patients with early OA, supporting the role of PFC‐FD as a safe nonoperative therapy to help prevent or delay the need for knee arthroplasty.

## AUTHOR CONTRIBUTIONS

**Tadahiko Ohtsuru**: Subject recruitment; data cleaning; clinical interpretation of results; writing; critical review of the manuscript. **Hiroto Hanai**: Subject recruitment; clinical interpretation of the results; critical review of the manuscript. **Taro Akimori**: Subject recruitment; clinical interpretation of the results; critical review of the manuscript. **Wataru Ando**: Subject recruitment; clinical interpretation of results; critical review of the manuscript. **Norimasa Nakamura**: Conceptualization; project development; clinical interpretation of the results; writing; critical review of the manuscript. **Stephen Lyman**: Conceptualization; study design; statistical analysis; writing; table creation; critical review of the manuscript. All authors approved this final version.

## FUNDING INFORMATION

CellSource, GK.

## CONFLICT OF INTEREST STATEMENT

Norimasa Nakamura receives research funding (for other projects) and holds stock options in CellSource, GK. Stephen Lyman serves as a methodological consultant and medical writer for CellSource, GK. The remaining authors declare no conflict of interest.

## ETHICS STATEMENT

This study was approved by the human research ethics committee of the Medical Corporation Katsujukai (approval number: 0006; Tokyo, Japan). Patients provided written consent to treatment with the clear acknowledgement that their clinical data would go into a research repository for future examination of clinical outcomes.

## Data Availability

The data that support the findings of this study may be available on request from the corresponding author. The data are not publicly available due to legal restrictions reflecting Japanese law regarding patient privacy.
